# Opportunities and Challenges in Developing a Cohort of Patients with Type 2 Diabetes Mellitus Using Electronic Primary Care Data

**DOI:** 10.1371/journal.pone.0162236

**Published:** 2016-11-18

**Authors:** Preeti Datta-Nemdharry, Andrew Thomson, Julie Beynon

**Affiliations:** Vigilance and Risk Management of Medicines (VRMM), MHRA, Victoria, London SW1W 9SZ, United Kingdom; Indiana University Richard M Fairbanks School of Public Health, UNITED STATES

## Abstract

**Purpose:**

To develop a cohort of patients with T2DM treated with insulin using CPRD to obtain an accurate diagnosis date. This was used to analyse time from T2DM diagnosis to first ever insulin prescription between 01/01/2000 and 30/06/2012, for patients in England and Wales.

**Methods:**

Patients aged 18 years and over at diagnosis, were included if prescribed an anti-diabetic drug and were excluded if first diagnosis-specific code was inconsistent with a T2DM diagnosis. Diagnosis codes were split into 8 categories based on whether they related to specific T2DM or non-specific diabetes codes. Patients were excluded if they had non-specific diagnosis codes and were prescribed insulin as their first-ever treatment for diabetes. Descriptive statistics for time from T2DM diagnosis to insulin initiation were calculated.

**Results:**

Two hundred and fifty-six codes were identified which were consistent with a first-ever diagnosis of T2DM. 7 codes were considered to clearly define a diagnosis of T2DM, which were reported for 64% of patients. The final cohort comprised 11,917 patients and the median time to first insulin prescription from the date of diagnosis was 4.4 years.

**Conclusions:**

A clear definition of cohort development is required to compare and interpret results from studies. Use of diagnosis and product codes is essential when examining use of drugs such as insulin, where competing diagnoses need to be considered separately.

## Introduction

The Clinical Practice Research Datalink (CPRD) is a longitudinal anonymised clinical database derived from approximately 650 primary care practices in the UK. It contains detailed information regarding demographic characteristics, clinical diagnoses, test results and prescriptions issued for patients in primary-care. The CPRD includes information from approximately 8% of the UK population and the practices included in CPRD constitute a broadly representative sample of all the practices in the UK [1].Due to the need for repeated long-term laboratory testing of patients with type 2 diabetes (T2DM), this database is a useful tool for studying patterns of disease and pharmacotherapy use in the primary-care setting. In the UK there are approximately 2.9 million people diagnosed with diabetes and an estimated 850,000 people with undiagnosed diabetes [2]. Around 90% of all adults with diabetes have T2DM [2] hence it is an important source of morbidity and mortality.

There are many challenges in developing a retrospective cohort of patients with T2DM from CPRD. In previous studies various criteria have been applied to identify patients with T2DM based on diagnostic record, prescription of different classes of anti-diabetic drugs or a combination of both [3–9].The objectives of most of these studies was to establish a cohort of patients with T2DM rather than to ascertain the date of T2DM diagnosis.

The objective of this paper is to describe the development of a cohort of patients with T2DM with an accurate date of first diagnosis from CPRD in order to analyse the time from diagnosis of T2DM to first ever prescription of insulin made between 01/01/2000 and 30/06/2012, for patients in England and Wales. This time frame of insulin initiation was chosen because this study was done to supplement a drug utilisation study examining patterns of anti-diabetic drug prescribing in CPRD from 01/01/2000 to 30/06/2012 (the latter study has been submitted for publication elsewhere). Since all patients received insulin the cohort does not reflect patients with T2DM who received other medications to manage glycaemia.

The main challenge lies in correctly identifying patients with T2DM, which involves a search using READ codes that refer to diagnosis of diabetes or related complications and examining the patients’ therapy records. Using appropriately inclusive search criteria, CPRD yields a large number of patients with diabetes-related READ codes, and each patient can have several records. The volume of data extracted means it is not feasible to analyse every record manually. However a purely computational approach is also not currently possible, due to the diversity of codes that are used.

## Methods

The cohort was initially identified using the CPRD product codes (based on Gemscript codes [10]) for anti-diabetic drugs. This constituted the therapy dataset and patients were included if they were prescribed at least one anti-diabetic drug.

After extracting data using product codes, the next step was to define the cohort by confirming the diagnosis of T2DM. The CPRD medical browser was used to search for broad terms related to diabetes and this yielded 599 medical codes (based on READ codes [11]). This included codes referring to Type 1 diabetes (T1DM), gestational diabetes, screening, family history of diabetes and other codes not relevant to T2DM. These codes were deleted leaving codes which were expected to include the majority of patients with T2DM. Hence a ‘top down’ approach was adopted where patients were included if they had been prescribed an anti-diabetic drug and in the next step were excluded if their first diagnosis code was considered inconsistent with a diagnosis of T2DM.

Following exclusion of irrelevant codes, 426 codes remained which were used to create a cohort of potential patients with T2DM. A variable denoting the first ever ‘event date’ relating to a diabetes code was created for each patient. If a patient’s first ever event date referring to a possible diagnosis of diabetes fell after the first ever prescription of anti-diabetic medication, the earlier time point (commencement of medication), was used.

The diagnosis codes were further split into 8 categories based on whether they related to a specific T2DM code or non-specific terms. The data were divided into 8 separate datasets following application of these criteria.

For patients with a non-specific diagnosis code for diabetes, the first prescription of an anti-diabetic drug was examined after merging the diagnosis and therapy datasets. Patients for whom the diagnosis of diabetes was not defined as either T1DM or T2DM, and who had been prescribed insulin as the first ever treatment for diabetes, were excluded from the study due to the likelihood of these patients having T1DM. The possibility of some of these excluded patients having T2DM is acknowledged, but this approach was considered less detrimental to the validity of the final cohort than including patients with an unclear diagnosis. All included patients had at least one year of up-to-standard follow-up prior to the first recorded diagnosis of diabetes in order to accurately define an incident cohort. Also patients should have had at most 1 gap in registration. The patients’ diagnosis date was after their registration date with the practice and they were followed until the GP’s last collection date, date of patient exiting the practice, date of death or end of study period. The final cohort comprised of patients with T2DM, aged 18 years and over at time of diagnosis, who were prescribed insulin in England and Wales.

### Analyses of time from diagnosis to insulin

The number of years from date of diabetes diagnosis to date of insulin initiation was calculated for the main insulin cohort and expressed as mean (95% confidence interval) and median (interquartile range).

Secondary analyses were performed as follows: i) with exclusion of patients who had a diabetes diagnosis date which was the same as the insulin initiation date ii) with exclusion of patients who had insulin prescribed as the first ever anti-diabetic drug iii) with inclusion of patients who were first diagnosed between 01/01/2000 and 30/06/2012. These were done to see what differences are observed when applying different exclusion criteria, as have been applied in other studies [3–9].

Analyses were conducted in STATA Version 11.

The study protocol was approved by the Independent Scientific Advisory Committee (ISAC) for MHRA Database Research and received no funding. CPRD is an anonymised electronic healthcare record database and hence further ethics approval is not required. Patient records are received in an anonymized and de-identified form for analysis.

## Results

### Formulation of cohort for time-to-event analysis

Of the 426 diagnostic codes that were used to create a cohort of patients with T2DM, there were 256 codes that constituted the first ever recorded diagnostic codes (Table A in S1 File). Initially patients whose first ever record of diagnosis was ‘diabetic screening’ were included. The total number of patients with a diabetes related code was 268,717, including all screening and diagnostic codes indicative of T2DM. However, diabetic screening codes were later deleted, because if a patient was subsequently diagnosed with diabetes (i.e. a patient had a relevant diabetes diagnosis code) then the diagnosis record would have been captured later on after deleting their initial screening code. Once screening codes were excluded, there were 267,783 patients remaining (this excluded patients who had no diabetes diagnostic code after an initial screening code).

Table 1 shows the percentage of patients belonging to each of the categories of diagnoses. 9.61% of patients had non-specific diagnosis codes, the inclusion or exclusion of which are discussed further below.

**Table 1:** Percentage of patients in the initial cohort by diagnostic category (total n = 267,783).

No.	Category of Diagnosis	% of total	Number of medical codes
1	T2DM^1^ specific	64.05	7
2	T2DM^1^ with complications, medications	0.92	51
3	DM^2^ specific	26.34	2
4	DM^2^ with complications, medications	2.54	39
5	Complication but no confirmed diagnosis mentioned; onset date likely to be earlier than the date of complication	1.11	61
6	Diagnosis and onset date not confirmed, but likely to be DM^2^	3.50	30
7	Diet	0.47	10
8	Other	1.07	56

^1:^ Type 2 diabetes mellitus

^2:^ Diabetes mellitus

The first two groups shown in Table 1 were included in the cohort. The third, fourth and seventh groups were included in the cohort if the first prescription for an anti-diabetic medication was not insulin (see List A in S1 File for details of the anti-diabetic drug classes). Diet-related medical codes implying underlying glucose intolerance or T2DM were included in this cohort with the associated diagnosis dates considered a proxy for the actual diagnosis dates on the notion that diet is usually first-line ‘therapy’ in the management of glycaemia in T2DM.

For the fifth, sixth and eighth categories it was difficult to accurately estimate the date of diagnosis of T2DM. The sixth group mainly consisted of patients with codes denoting diabetic monitoring or diabetic review. While almost certain to have had a preceding diagnosis of diabetes, no information was available on the date of first diagnosis. Group 8, which is described as ‘other’, contained a variety of different codes ranging from diabetes education-related codes to diabetes referral-related codes. For the purpose of time-to-event analyses groups 5, 6 and 8 were excluded.

Table 2 summarises the decision made and how the size of the diagnosis cohort varied following application of different decisions.

**Table 2:** Decisions made in formulating initial diagnosis dataset and impact on sample size.

No.	Diagnosis	Decision: Include/Exclude	Cumulative size
1	Specific T2DM^1^	Include	171,504
2	T2DM^1^ with Complications, medications	Include	173,958
3	Specific DM^2^	Include, if first prescription was not insulin	244,492
4	DM^2^ with Complications, medications	Include, if first prescription was not insulin	251,291
5	Complication but no confirmed diagnosis mentioned, onset date likely to be earlier than this	Exclude	251,291
6	Diagnosis and onset date not confirmed, but likely to be DM^2^	Exclude	251,291
7	Diet	Include, if first prescription was not insulin	252,558
8	Other	Exclude	252,558

^1:^ Type 2 diabetes mellitus

^2:^ Diabetes mellitus

**Fig 1:**
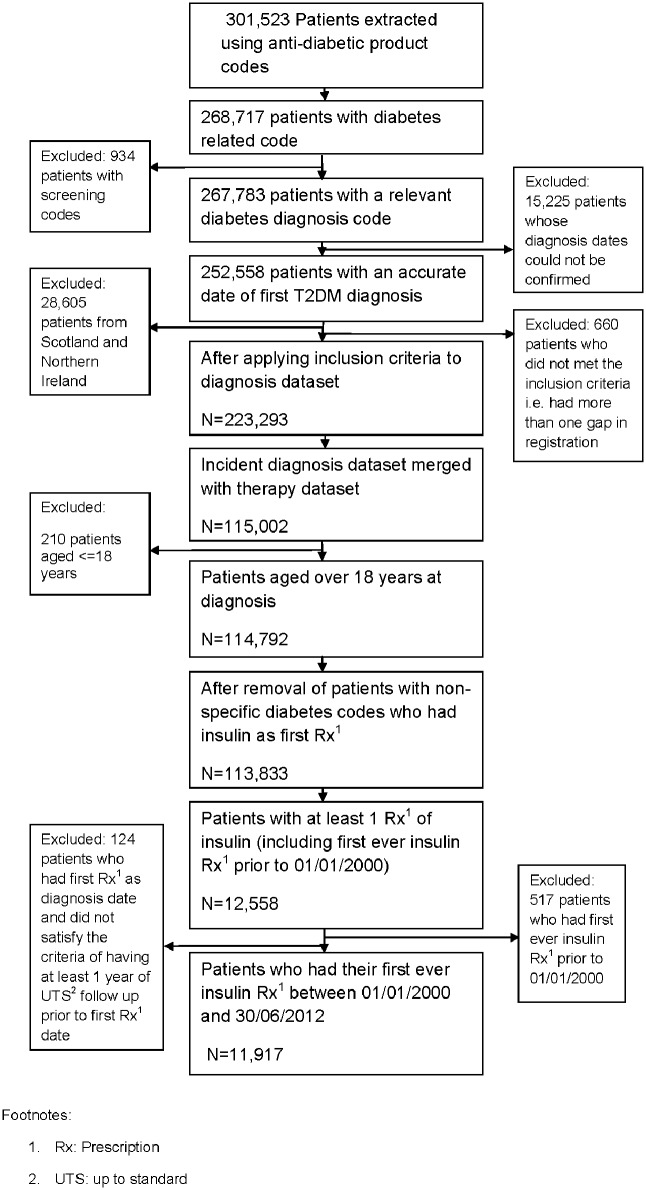
Flowchart of patients included in the cohort.

Fig 1 shows a flowchart of patients included in the cohort. Once the initial diagnosis dataset was finalised this was merged with the therapy dataset. The final cohort consisted of 11,917 patients who had their first ever insulin prescription between 01/01/2000 and 30/06/2012. Around 91% of the patients in the cohort were aged 40 or over and 56% were men. Sixty-five per cent of patients had a T2DM-specific diagnosis code.

### Time from diagnosis of T2DM to the first insulin prescription

A positively skewed distribution was observed for time from diagnosis to insulin prescription with a median time of 4.41 years (IQR 1.66 to 7.56years) for patients with first ever insulin prescription between 01/01/2000 and 30/06/2012. Around 15% of patients (n = 1805) in the cohort were prescribed their first ever insulin prescription up to 6 months after the diagnosis date. Approximately 2.3% of patients in the cohort had specific T2DM codes and a first ever anti-diabetic drug prescription of insulin at time of diagnosis. When the analysis was limited to patients aged 40 and over in the cohort the median time from diagnosis to first insulin prescription increased slightly (4.57 years for patients aged 40 and over vs. 4.4 years for patients aged 18 and over). When patients whose first anti-diabetic prescription date was taken as the diagnosis date (n = 730; 6% of cohort) were removed from the analysis the median time from diagnosis to first insulin prescription was 4.57 years.

The results for time to first prescription of insulin by the patients’ baseline characteristics are shown in Table 3.

**Table 3:** Time (years) from diagnosis of T2DM to first insulin prescription (between 01/01/2000 and 30/06/2012) by baseline characteristics.

	Number of patients	Number of years from date of diagnosis to date of first insulin prescription for all patients with insulin	
	N (%)	Mean (95% CI)	Median (IQR)
Gender:			
Male	6697 (56.2)	5.09 (4.99, 5.19)	4.55 (1.65, 7.76)
Female	5220 (43.8)	4.87 (4.76, 4.97)	4.26 (1.68, 7.32)
Age group at diagnosis (years):			
18–29	191 (1.60)	3.05 (2.63, 3.46)	2.26 (0.66, 4.73)
30–39	884 (7.42)	4.07 (3.82, 4.32)	3.10 (0.95, 6.28)
40–49	2209 (18.54)	5.07 (4.91, 5.24)	4.51 (1.82, 7.54)
50–59	3311 (27.78)	5.40 (5.26, 5.53)	4.90 (2.17, 8.02)
60–69	3057 (25.65)	5.46 (5.31, 5.61)	5.02 (2.15, 8.20)
70–79	1793 (15.05)	4.60 (4.43, 4.78)	4.12 (1.25, 7.19)
80–99	472 (3.96)	2.77(2.52, 3.02)	1.95(0.25, 4.54)
Year of first ever diagnosis			
≤1995	1153 (9.68)	11.14 (10.94, 11.34)	10.74 (8.46, 13.51)
1996–1997	821 (6.89)	8.31 (8.09, 8.53)	8.03 (5.72, 10.62)
1998–1999	1323 (11.10)	6.71 (6.54, 6.89)	6.47 (4.04, 9.16)
2000	977 (8.20)	5.89 (5.67, 6.10)	5.82 (3.23, 4.49)
2001	1060 (8.89)	5.35 (5.16, 5.54)	5.22 (2.80, 7.93)
2002	1102 (9.25)	4.68 (4.51, 4.85)	4.53 (2.19, 7.03)
2003	1007 (8.45)	4.11 (3.94, 4.28)	4.01 (1.63, 6.51)
2004	877 (7.36)	3.70 (3.54, 3.87)	3.70 (1.47, 5.75)
2005	793 (6.65)	3.25 (3.09, 3.41)	3.26 (1.07, 5.22)
2006	727 (6.10)	2.36 (2.22, 2.50)	2.03 (0.44, 4.06)
2007	608 (5.10)	2.01 (1.88, 2.15)	1.79 (0.16, 3.59)
2008	521 (4.27)	1.56 (1.44, 1.67)	1.25 (0.17, 2.82)
2009	415 (3.48)	0.94 (0.84, 1.04)	0.46 (0.05, 1.72)
2010	286 (2.40)	0.71 (0.61, 0.80)	0.28 (0.04, 1.32)
2011	186 (1.56)	0.30 (0.24, 0.35)	0.11 (0.02, 0.51)
2012*	61 (0.51)	0.12 (0.08, 0.15)	0.06 (0.01, 0.16)
Year of first ever prescription of insulin			
2000	353 (2.96)	3.72(3.43, 4.02)	3.34(1.19, 5.81)
2001	444 (3.73)	3.89(3.61, 4.18)	3.38 (1.13, 6.15)
2002	543 (4.56)	3.85(3.59, 4.12)	3.13 (1.13, 6.49)
2003	747 (6.27)	3.85(3.61, 4.09)	3.12 (1.04, 5.96)
2004	968 (8.12)	4.33(4.10, 4.56)	3.51 (1.43, 6.45)
2005	1008 (8.46)	4.76(4.53, 4.98)	4.20 (1.79, 7.03)
2006	1077 (9.04)	4.47(4.25, 4.68)	3.89 (1.46, 6.50)
2007	1191 (9.99)	4.71(4.49, 4.93)	4.30 (1.18, 7.07)
2008	1190 (9.99)	5.19(4.96, 5.42)	4.73 (1.88, 7.74)
2009	1191 (9.99)	5.22(4.98, 5.46)	4.85 (1.38, 7.95)
2010	1171 (9.83)	5.65(5.40, 5.90)	5.20 (1.95, 8.53)
2011	1177 (9.88)	6.16(5.92, 6.41)	5.93 (2.82, 9.09)
2012*	857 (7.19)	6.77(6.47, 7.07)	6.55 (3.11, 9.97)

*till 30/06/2012

IQR: Interquartile range

The median number of years from date of T2DM diagnosis to date of first insulin was 4.55 years for men as compared to 4.26 years for women, a difference of 4 months. Patients aged between 30 to 39 years had a lower median time until start of insulin in comparison to those aged 40 or over. There was a steady increase in time to insulin prescription as the year of insulin initiation increased (median of 3.34 years in 2000 vs. 5.93 years in 2011).

Other anti-diabetic medications taken by patients before they started insulin were also looked at. Table 4 shows the most common patterns of therapy/anti-diabetic drugs prescribed (≥100 patients per pathway) prior to insulin initiation (the patients could have had other antidiabetic therapy after insulin; however this is not shown in the table as we are interested in time to first prescription of insulin). Patients could have had concomitant therapies in their pathways (including at first ever prescription) which is not shown in Table 4. The number of patients in Table 4 constitutes around 80% (n = 9,514) of the whole cohort.

**Table 4:** The most common anti-diabetic drug pathways prior to insulin initiation.

Patterns	N (% of total cohort n = 11,917)	Time (years) from diagnosis of T2DM to insulin initiation	
		Mean (95% CI)	Median (IQR)
Met, Sulph, Insulin	2317 (19.44)	4.59 (4.46, 4.72)	4.03 (2.02, 6.55)
Sulph, Met, Insulin	1529(12.83)	6.20 (6.02, 6.39)	5.88 (3.24, 8.68)
Insulin*	1167(9.79)	0.56 (0.47, 0.64)	0.04 (0.003, 0.20)
Met, Sulph, TZD, Insulin	934 (7.84)	6.20 (5.97, 6.43)	5.66 (3.49, 8.41)
Met, Insulin	914(7.67)	2.27 (2.10, 2.44)	1.27 (0.24, 3.50)
Sulph, Insulin	826 (6.93)	2.87 (2.66, 3.08)	1.85 (0.32, 4.31)
Sulph, Met, TZD, Insulin	771 (6.47)	7.72 (7.46, 7.99)	7.59 (4.84, 10.38)
Met, Sulph, DPP-4 inhibitors, Insulin	313 (2.63)	6.12 (5.73, 6.50)	5.60 (3.62, 7.42)
Met, TZD, Sulph, Insulin	275 (2.31)	5.58 (5.21, 5.96)	5.17 (3.20, 7.42)
Met, TZD, Insulin	248 (2.08)	4.20 (3.82, 4.58)	3.49 (1.87, 5.84)
Sulph, TZD, Insulin	111 (0.93)	4.86 (4.17, 5.55)	4.25 (1.99, 6.39)
Met, Sulph, TZD, DPP-4 inhibitors, Insulin	109 (0.91)	8.22 (7.48, 8.96)	7.98 (5.49, 10.82)

Note: Patients could have had concomitant therapies in their pathways (including at first ever prescription) which is not shown in Table 4

IQR: Interquartile range

Met: Metformin; Sulph: Sulphonylureas; TZD: Thiazolidinediones

*These patients had insulin as their first ever prescription. Insulin includes human insulin, insulin analogue and animal insulin.

Of the patients in Table 4, 7410 (78%) had metformin at some stage in their treatment pathway and 7185 (75%) had sulphonylurea at some stage in their treatment pathway.

For pathways followed by between 10 and 99 patients i.e. not included in Table 4 (n = 1,705; 14.30% of the cohort) the mean time to diagnosis was in the range of 3.21 years to 12.07 years.

Excluding patients with a questionable diagnosis of T2DM (i.e. those who had same insulin prescription and diagnosis dates or those whose first ever anti-diabetic drug was insulin) increased the average values for time to first prescription of insulin by a few months (Table 5).

**Table 5:** Summary statistics of time (years) from diagnosis of T2DM to date of first insulin prescription (between 01/01/2000 and 30/06/2012).

	Number of patients	Number of years from diagnosis to date of first insulin prescription	
	N	Mean (95% CI)	Median (IQR*)
i) All patients with insulin	11,917	4.99 (4.92, 5.06)	4.41 (1.66, 7.56)
ii) After deleting patients with first insulin prescription date same as diagnosis date	11,641	5.11 (5.04, 5.18)	4.54 (1.86, 7.66)
iii) After deleting patients with insulin as first prescription after diagnosis	10,750	5.47 (5.40, 5.55)	4.95 (2.39, 7.95)
iv) Including patients who were first diagnosed between 01/01/2000 and 30/06/2012	8,643	3.59 (3.53, 3.66)	3.11 (0.85, 5.69)

*Interquartile range

## Discussion

This paper has described the development of a cohort of patients with T2DM in CPRD. We used this to analyse time from T2DM diagnosis to insulin prescription for patients with first ever insulin prescription between 01/01/2000 and 30/06/2012. Previous studies using CPRD have developed cohorts of patients with T2DM based on diagnosis code, pharmacological intervention or both and have addressed questions regarding associations between various anti-diabetic drugs and various outcomes including mortality and cancer [4–9, 12,13]. Initially, in the diagnosis dataset, around ten per cent of patients had non-specific diagnoses for T2DM; the categories of codes were assessed further and some of them deleted if it was not possible to get an accurate date of diagnosis based on the information available in the text of the codes. It was important to examine these further as failure to include these might have led to an unrepresentative cohort, discussed further below.

From 2006 onwards we observed an increase in the median time to first insulin prescription. This has also been observed in a study using the Disease Analyzer database [14] in Germany and UK, although the median time to insulin by year in this study was slightly longer than that observed in our study. This may be due to the authors having excluded patients with the same dates for first prescription of insulin and diabetes diagnosis. In our study median time to insulin initiation increased by 2 months when we excluded patients with the same dates for insulin prescription and diabetes diagnosis. The steady increase in time to insulin prescription as the year of initiation increased may reflect recent developments in pharmacotherapy of T2DM and greater awareness of the importance of diet and lifestyle factors. The introduction of novel anti-diabetic agents such as DPP-4 inhibitors and GLP-1 agonists in 2006 and 2007 respectively might have potentially raised the line of insulin therapy.

A study [15] using GPRD (precursor of CPRD) reported time to insulin initiation in patients with T2DM who were inadequately controlled on oral glucose lowering agents (OGLA). The authors of this study estimated that around 25% of patients who had an HbA1C above the OGLA threshold of > = 8% would initiate insulin within 1.8 years of OGLA failure, and 50% of patients within 4.9 years. While identifying patients who fail on OGLAs is one way of developing a cohort of insulin users with T2DM, this approach will exclude patients with T2DM who progress to insulin prior to use or failure of two OGLAs.

### Opportunities and Challenges

The data from CPRD enabled the development of a cohort of patients with type 2 diabetes. CPRD is a useful resource of primary care data and provides the opportunity to study patients with different diagnoses. For this study, the diagnosis and therapy datasets were combined to form a cohort for analysis of time to insulin initiation. This meant that, to be included, a patient required an accurate diagnosis of diabetes and at least one prescription of insulin. The inclusion of patients with relevant categories of diagnosis codes optimized the size of the cohort of patients with T2DM. A simple coding of T2DM, for example, only identified 65% of the final cohort used for the analyses. It also allows a broader spectrum of patients with T2DM to be included, including those identified early and late in the disease process. The diagnosis code could contain further information related to disease. For example, a code of T2DM with complications may imply a patient is at a later stage in the disease process. Such group of patients may be likely to be prescribed insulin more quickly if their T2DM is not well-controlled.

Given the acknowledged limitations of CPRD, the challenge was to ensure that the cohort was defined as accurately as possible. It was unfeasible to pre-specify all search criteria and classification decisions until after the initial data extraction. The categories used to define the data were arbitrary. Additionally, date of diagnosis could not be estimated for the whole of the initial patient cohort as there was uncertainty regarding some codes e.g. patients whose first ever diagnosis was diabetic retinopathy. This led to exclusion of some patients with a likely longer duration of T2DM prior to formal diagnosis and may have affected the results of the time to insulin initiation analysis. One study suggests that up to 35% of newly diagnosed patients with T2DM have retinopathy symptoms and diagnosis can occur late in the disease process in the UK [16].

In our study those patients who had ‘diet’ as a treatment code were included since diet is used to control glycaemia prior to the start of any pharmacological interventions [17] although this might include patients with impaired glucose tolerance who do not have confirmed T2DM. Patients who had codes related to ‘T2DM with complications’ and ‘DM with complications’ were included in the cohort but are likely to have had diabetes for longer. This may have led to an underestimate in the time to first insulin prescription.

In order to exclude patients with T1DM, earlier studies have applied the approach of deleting patients if their first ever anti-diabetic drug was insulin [3, 4] based on the assumption that those patients who started treatment with insulin are more likely to have T1DM. In our study we applied an additional criterion where patients’ records were deleted if their first ever anti-diabetic drug after diagnosis was insulin and the record was not specific with respect to T2DM diagnosis. Nevertheless, there is a possibility that patients with advanced T2DM at diagnosis may have been prescribed insulin as first-line therapy. After deleting 10% of patients with a specific T2DM diagnosis and who had insulin prescribed as first-line therapy, the median time to first insulin prescription increased by around 6 months. Furthermore, not all patients had a diagnosis code and misclassification of diagnosis is a possibility. The lower median time until start of insulin observed in patients aged 18 to 39 years compared to those aged 40 years and over might mean that some patients with T1DM were misclassified as patients with T2DM although it may also suggest those with early-onset disease progress faster. However, restricting the cohort to those aged 40 years and over showed that patients in this group had a median difference of around 2 months greater than in the cohort of patients aged 18 and over reflecting only a minor increase.

Further interrogation of individual records might allow validation of cases where the diagnosis of T2DM is not confirmed, but such work is beyond the scope of this paper. External validation of diagnosis codes from CPRD with additional information from GP practices has been conducted for other studies, such as those involving diagnoses of rheumatoid arthritis and juvenile idiopathic arthritis [18]. However, such work depends on the resource available.

### Wider Implications

It is important to consider how screening codes, as opposed to more explicit diagnostic codes, are handled in accurately identifying a cohort of patients with T2DM. In our approach screening codes were deleted and patients with a subsequent diagnosis code were included. If glucose intolerance has been identified early and is closely monitored by the GP, a patient may have a different treatment pathway and achieve better long-term outcomes than a patient with a specific T2DM diagnosis. This is important to examine when considering associations between medications and outcomes. Similarly, patients who had codes in the categories of ‘T2DM with complications’ and ‘DM with complications’ were included in the cohort but are likely to have 1) different characteristics than those with a specific T2DM diagnosis 2) different treatment pathways and 3) a larger influence in subsequent studies.

## Conclusions

This paper has highlighted the advantages and challenges faced when using both diagnosis and therapy data to establish the date of diagnosis of T2DM in a retrospective cohort in CPRD. Such an approach is essential when examining use of drugs such as insulin, where competing diagnoses (T1DM and T2DM) need to be considered separately. To ensure that results obtained from different cohort studies can be compared and interpreted, a clear definition of cohort development is required. It also acts as a reference for studies in a different database and for developing different disease cohorts in the same database. Although far more challenging to construct, cohorts based on diagnoses, rather than on treatments alone, potentially include more accurate and informative data and allow an array of suitable sensitivity analyses to be conducted.

## Supporting Information

S1 File **Table A:** in CPRD medical codes for first ever recorded diabetes diagnosis codes. **List A:** Different classes of anti-diabetic medications.
(DOC)
