# Supplementary material for: Opportunities and Challenges in Developing a Cohort of Patients with Type 2 Diabetes Mellitus Using Electronic Primary Care Data
Source: PLoS One. 2016 Nov 18;11(11):e0162236. doi: 10.1371/journal.pone.0162236 (PMC5115653; doi:10.1371/journal.pone.0162236)
Supplement: S1 File — Table A: in CPRD medical codes for first ever recorded diabetes diagnosis codes. List A: Different classes of anti-diabetic medications. (DOC) [file pone.0162236.s001.doc]

**S1 FILE**

**Table A: CPRD medical codes for first ever recorded diabetes diagnosis codes**

| Medical code | Description | No. of patients | % | Categories |
| --- | --- | --- | --- | --- |
| 506 | Non-insulin dependent diabetes mellitus | 9,878 | 3.69 | 1 |
| 758 | Type 2 diabetes mellitus | 139,909 | 52.25 | 1 |
| 4513 | Non-insulin dependent diabetes mellitus | 11,606 | 4.33 | 1 |
| 5884 | NIDDM - Non-insulin dependent diabetes mellitus | 940 | 0.35 | 1 |
| 17859 | Type 2 diabetes mellitus | 7,807 | 2.92 | 1 |
| 18219 | Type II diabetes mellitus | 751 | 0.28 | 1 |
| 22884 | Type II diabetes mellitus | 613 | 0.23 | 1 |
| 1407 | Insulin treated Type 2 diabetes mellitus | 681 | 0.25 | 2 |
| 8403 | Non-insulin dependent diabetes mellitus - poor control | 4 | 0.00 | 2 |
| 12640 | Type 2 diabetes mellitus with nephropathy | 13 | 0.00 | 2 |
| 12736 | Type 2 diabetes mellitus with gangrene | 1 | 0.00 | 2 |
| 14803 | Diabetes mellitus, adult onset, no mention of complication | 467 | 0.17 | 2 |
| 14889 | Maturity onset diabetes | 910 | 0.34 | 2 |
| 17262 | Non-insulin-dependent diabetes mellitus with retinopathy | 2 | 0.00 | 2 |
| 18209 | Type 2 diabetes mellitus with renal complications | 1 | 0.00 | 2 |
| 18264 | Insulin treated Type II diabetes mellitus | 1 | 0.00 | 2 |
| 18278 | Insulin treated Type 2 diabetes mellitus | 105 | 0.04 | 2 |
| 18390 | Type 2 diabetes mellitus with persistent microalbuminuria | 19 | 0.01 | 2 |
| 18425 | Type 2 diabetes mellitus with polyneuropathy | 3 | 0.00 | 2 |
| 18496 | Type 2 diabetes mellitus with retinopathy | 22 | 0.01 | 2 |
| 18777 | Type 2 diabetes mellitus with renal complications | 4 | 0.00 | 2 |
| 25627 | Type 2 diabetes mellitus - poor control | 10 | 0.00 | 2 |
| 26054 | Type 2 diabetes mellitus with persistent proteinuria | 4 | 0.00 | 2 |
| 29979 | Non-insulin-dependent diabetes mellitus without complication | 21 | 0.01 | 2 |
| 32627 | Type 2 diabetes mellitus with ketoacidosis | 25 | 0.01 | 2 |
| 34268 | Type 2 diabetes mellitus with neurological complications | 1 | 0.00 | 2 |
| 34912 | Non-insulin dependent diabetes mellitus with ulcer | 1 | 0.00 | 2 |
| 35385 | Type 2 diabetes mellitus with neuropathic arthropathy | 1 | 0.00 | 2 |
| 37806 | Type 2 diabetes mellitus with peripheral angiopathy | 2 | 0.00 | 2 |
| 41389 | Diabetes mellitus, adult onset, + ophthalmic manifestation | 2 | 0.00 | 2 |
| 44982 | Type 2 diabetes mellitus with diabetic cataract | 1 | 0.00 | 2 |
| 45919 | Type 2 diabetes mellitus with neurological complications | 1 | 0.00 | 2 |
| 46624 | Maturity onset diabetes in youth | 7 | 0.00 | 2 |
| 46917 | Type 2 diabetes mellitus with hypoglycaemic coma | 3 | 0.00 | 2 |
| 47315 | Type II diabetes mellitus - poor control | 7 | 0.00 | 2 |
| Medical code | Description | No. of patients | % | Categories |
| 47321 | Type 2 diabetes mellitus with ophthalmic complications | 3 | 0.00 | 2 |
| 47954 | Type 2 diabetes mellitus without complication | 58 | 0.02 | 2 |
| 49074 | Type 2 diabetes mellitus with ulcer | 2 | 0.00 | 2 |
| 49655 | Type II diabetes mellitus with retinopathy | 2 | 0.00 | 2 |
| 50225 | Type II diabetes mellitus with renal complications | 1 | 0.00 | 2 |
| 50429 | Non-insulin-dependent diabetes mellitus with ophthalm comps | 1 | 0.00 | 2 |
| 50527 | Type II diabetes mellitus with polyneuropathy | 1 | 0.00 | 2 |
| 50609 | Pre-existing diabetes mellitus, non-insulin-dependent | 2 | 0.00 | 2 |
| 51756 | Type 2 diabetes mellitus with ketoacidotic coma | 4 | 0.00 | 2 |
| 52303 | Non-insulin-dependent diabetes mellitus with renal comps | 2 | 0.00 | 2 |
| 53392 | Type II diabetes mellitus without complication | 26 | 0.01 | 2 |
| 58604 | Type II diabetes mellitus with retinopathy | 2 | 0.00 | 2 |
| 59253 | Type 2 diabetes mellitus with arthropathy | 2 | 0.00 | 2 |
| 59365 | Non-insulin dependent diabetes mellitus with nephropathy | 1 | 0.00 | 2 |
| 59991 | Maturity onset diabetes in youth type 2 | 2 | 0.00 | 2 |
| 62146 | Non-insulin-dependent diabetes mellitus with multiple comps | 1 | 0.00 | 2 |
| 62674 | Type 2 diabetes mellitus with mononeuropathy | 2 | 0.00 | 2 |
| 63690 | Type 2 diabetes mellitus with gastroparesis | 1 | 0.00 | 2 |
| 63762 | Diabetes mellitus, adult onset, + unspecified complication | 5 | 0.00 | 2 |
| 64668 | Insulin treated Type II diabetes mellitus | 14 | 0.01 | 2 |
| 65267 | Type 2 diabetes mellitus with multiple complications | 1 | 0.00 | 2 |
| 91646 | Type II diabetes mellitus with ulcer | 1 | 0.00 | 2 |
| 98723 | Type II diabetes mellitus with hypoglycaemic coma | 1 | 0.00 | 2 |
| 711 | Diabetes mellitus | 70,524 | 26.34 | 3 |
| 52212 | [X]Diabetes mellitus | 10 | 0.00 | 3 |
| 1684 | Diabetic on oral treatment | 971 | 0.36 | 4 |
| 2378 | Diabetic - poor control | 298 | 0.11 | 4 |
| 2379 | Seen in diabetic clinic | 3,803 | 1.42 | 4 |
| 7563 | Diabetic on diet only | 777 | 0.29 | 4 |
| 7795 | Diabetes mellitus with neuropathy | 42 | 0.02 | 4 |
| 8842 | Diabetic on insulin | 145 | 0.05 | 4 |
| 9974 | Seen in diabetic eye clinic | 77 | 0.03 | 4 |
| 10824 | Seen in diabetic foot clinic | 35 | 0.01 | 4 |
| 13069 | Has seen dietician - diabetes | 80 | 0.03 | 4 |
| 13071 | Diabetic - good control | 196 | 0.07 | 4 |
| 13078 | Diabetic weight reducing diet | 88 | 0.03 | 4 |
| 16230 | Diabetes mellitus with neurological manifestation | 6 | 0.00 | 4 |
| 16491 | Diabetes mellitus with polyneuropathy | 1 | 0.00 | 4 |
| 16502 | Diabetes mellitus with renal manifestation | 2 | 0.00 | 4 |
| 16946 | Diabetic child | 2 | 0.00 | 4 |
| Medical code | Description | No. of patients | % | Categories |
| 17869 | Diabetic-uncooperative patient | 1 | 0.00 | 4 |
| 21482 | Diabetes mellitus with hyperosmolar coma | 4 | 0.00 | 4 |
| 22023 | Diabetic - poor control NOS | 1 | 0.00 | 4 |
| 28769 | Diabetic on insulin and oral treatment | 3 | 0.00 | 4 |
| 32556 | Diabetes with gangrene | 1 | 0.00 | 4 |
| 32739 | Seen in community diabetes specialist clinic | 3 | 0.00 | 4 |
| 33254 | Diabetes mellitus with ophthalmic manifestation | 11 | 0.00 | 4 |
| 33343 | Diabetes mellitus with other specified manifestation | 1 | 0.00 | 4 |
| 35321 | Non-urgent diabetic admission | 2 | 0.00 | 4 |
| 35383 | Diabetic patient unsuitable for digital retinal photography | 6 | 0.00 | 4 |
| 35399 | Diabetes mellitus with peripheral circulatory disorder | 2 | 0.00 | 4 |
| 36695 | Diabetes mellitus autosomal dominant type 2 | 26 | 0.01 | 4 |
| 38103 | Seen in diabetic nurse consultant clinic | 3 | 0.00 | 4 |
| 38129 | Seen in community diabetic specialist nurse clinic | 1 | 0.00 | 4 |
| 38986 | Diabetes mellitus with no mention of complication | 71 | 0.03 | 4 |
| 43453 | Diabetes mellitus autosomal dominant | 2 | 0.00 | 4 |
| 43951 | Diabetic - cooperative patient | 3 | 0.00 | 4 |
| 45491 | Diabetes mellitus with unspecified complication | 2 | 0.00 | 4 |
| 46521 | Seen by diabetologist | 22 | 0.01 | 4 |
| 50972 | Diabetes mellitus NOS with no mention of complication | 13 | 0.00 | 4 |
| 55431 | Pre-existing diabetes mellitus, unspecified | 2 | 0.00 | 4 |
| 64357 | Diabetes mellitus NOS with unspecified complication | 7 | 0.00 | 4 |
| 72345 | Diabetes mellitus NOS with hyperosmolar coma | 2 | 0.00 | 4 |
| 102316 | Suspected diabetes mellitus | 87 | 0.03 | 4 |
| 1323 | Diabetic retinopathy | 380 | 0.14 | 5 |
| 2340 | Diabetic amyotrophy | 6 | 0.00 | 5 |
| 2342 | Diabetic neuropathy | 36 | 0.01 | 5 |
| 2471 | Nephrotic syndrome in diabetes mellitus | 2 | 0.00 | 5 |
| 2475 | Diabetic nephropathy | 10 | 0.00 | 5 |
| 2986 | Preproliferative diabetic retinopathy | 3 | 0.00 | 5 |
| 3286 | Proliferative diabetic retinopathy | 17 | 0.01 | 5 |
| 3837 | Diabetic maculopathy | 57 | 0.02 | 5 |
| 5002 | Diabetic polyneuropathy | 4 | 0.00 | 5 |
| 6813 | H/O: diabetes mellitus | 1,734 | 0.65 | 5 |
| 7059 | Admit diabetic emergency | 21 | 0.01 | 5 |
| 7069 | Background diabetic retinopathy | 133 | 0.05 | 5 |
| 7328 | Cellulitis in diabetic foot | 2 | 0.00 | 5 |
| 9835 | O/E - diabetic maculopathy present both eyes | 6 | 0.00 | 5 |
| 9881 | Mixed diabetic ulcer - foot | 8 | 0.00 | 5 |
| 10099 | Advanced diabetic maculopathy | 8 | 0.00 | 5 |
| 10659 | Diabetic cataract | 7 | 0.00 | 5 |
| 10755 | Non proliferative diabetic retinopathy | 18 | 0.01 | 5 |
| 11129 | O/E - left eye background diabetic retinopathy | 30 | 0.01 | 5 |
| Medical code | Description | No. of patients | % | Categories |
| 11433 | O/E - right eye background diabetic retinopathy | 50 | 0.02 | 5 |
| 11599 | Pan retinal photocoagulation for diabetes | 7 | 0.00 | 5 |
| 11626 | Diabetic retinopathy NOS | 14 | 0.01 | 5 |
| 11663 | Neuropathic diabetic ulcer - foot | 9 | 0.00 | 5 |
| 12247 | Diabetic foot examination not indicated | 19 | 0.01 | 5 |
| 13097 | O/E - right eye proliferative diabetic retinopathy | 2 | 0.00 | 5 |
| 13099 | O/E - right eye preproliferative diabetic retinopathy | 2 | 0.00 | 5 |
| 13100 | O/E - no right diabetic retinopathy | 53 | 0.02 | 5 |
| 13101 | O/E - left eye proliferative diabetic retinopathy | 5 | 0.00 | 5 |
| 13102 | O/E - right eye diabetic maculopathy | 15 | 0.01 | 5 |
| 13103 | O/E - left eye preproliferative diabetic retinopathy | 1 | 0.00 | 5 |
| 13104 | O/E - no left diabetic retinopathy | 53 | 0.02 | 5 |
| 13108 | O/E - left eye diabetic maculopathy | 10 | 0.00 | 5 |
| 17067 | Autonomic neuropathy due to diabetes | 9 | 0.00 | 5 |
| 17095 | O/E - Right diabetic foot at risk | 5 | 0.00 | 5 |
| 17247 | Diabetic mononeuritis NOS | 2 | 0.00 | 5 |
| 18056 | Foot abnormality - diabetes related | 1 | 0.00 | 5 |
| 18142 | Diabetic cheiroarthropathy | 1 | 0.00 | 5 |
| 18824 | Diabetic foot examination declined | 4 | 0.00 | 5 |
| 20696 | Injection sites - diabetic | 1 | 0.00 | 5 |
| 22823 | Diabetic foot examination | 27 | 0.01 | 5 |
| 22967 | Retinal abnormality - diabetes related | 2 | 0.00 | 5 |
| 24327 | Ischaemic ulcer diabetic foot | 8 | 0.00 | 5 |
| 24571 | Asymptomatic diabetic neuropathy | 1 | 0.00 | 5 |
| 26664 | O/E - Left diabetic foot at risk | 1 | 0.00 | 5 |
| 26666 | O/E - Right diabetic foot at low risk | 58 | 0.02 | 5 |
| 26667 | O/E - Left diabetic foot at low risk | 59 | 0.02 | 5 |
| 27891 | Diabetic Charcot arthropathy | 3 | 0.00 | 5 |
| 31156 | O/E - Left diabetic foot at moderate risk | 16 | 0.01 | 5 |
| 31157 | O/E - Right diabetic foot at moderate risk | 18 | 0.01 | 5 |
| 31171 | O/E - Right diabetic foot at high risk | 7 | 0.00 | 5 |
| 31172 | O/E - Left diabetic foot at high risk | 5 | 0.00 | 5 |
| 31790 | Polyneuropathy in diabetes | 5 | 0.00 | 5 |
| 34152 | Diabetic peripheral angiopathy | 1 | 0.00 | 5 |
| 35116 | O/E - Left diabetic foot - ulcerated | 2 | 0.00 | 5 |
| 35316 | O/E - Right diabetic foot - ulcerated | 2 | 0.00 | 5 |
| 35785 | Chronic painful diabetic neuropathy | 3 | 0.00 | 5 |
| 37315 | Diabetic mononeuropathy | 3 | 0.00 | 5 |
| 47144 | O/E - diabetic maculopathy absent both eyes | 15 | 0.01 | 5 |
| 49640 | O/E - left chronic diabetic foot ulcer | 1 | 0.00 | 5 |
| 53634 | [D]Gangrene of toe in diabetic | 2 | 0.00 | 5 |
| 68928 | Adverse reaction to insulins and antidiabetic agents | 1 | 0.00 | 5 |
| 3550 | Diabetic monitoring | 3,113 | 1.16 | 6 |
| 6125 | Diabetic annual review | 1,122 | 0.42 | 6 |
| 6430 | Attending diabetes clinic | 117 | 0.04 | 6 |
| 8836 | Diabetes management plan given | 162 | 0.06 | 6 |
| Medical code | Description | No. of patients | % | Categories |
| 9897 | Diabetes monitoring admin. | 3,310 | 1.24 | 6 |
| 11471 | Diabetes medication review | 39 | 0.01 | 6 |
| 12030 | Diabetes monitoring 3rd letter | 2 | 0.00 | 6 |
| 12213 | Patient on maximal tolerated therapy for diabetes | 3 | 0.00 | 6 |
| 12307 | Diabetes care by hospital only | 27 | 0.01 | 6 |
| 13067 | Diabetic monitoring NOS | 348 | 0.13 | 6 |
| 13191 | Diabetes clinic administration | 12 | 0.00 | 6 |
| 13192 | Diabetes monitor. check done | 204 | 0.08 | 6 |
| 13194 | Diabetes monitoring 1st letter | 418 | 0.16 | 6 |
| 13195 | Diabetes monitoring 2nd letter | 8 | 0.00 | 6 |
| 13197 | Attends diabetes monitoring | 261 | 0.10 | 6 |
| 16490 | Diabetic treatment changed | 5 | 0.00 | 6 |
| 20900 | Diabetes monitored | 30 | 0.01 | 6 |
| 22130 | Diabetes monitoring default | 18 | 0.01 | 6 |
| 24363 | Diabetic stabilisation | 24 | 0.01 | 6 |
| 28873 | Diabetic 6 month review | 36 | 0.01 | 6 |
| 29041 | Date diabetic treatment start | 29 | 0.01 | 6 |
| 31141 | Diabetes monitor.phone invite | 30 | 0.01 | 6 |
| 31240 | Diabetes monitor.verbal invite | 33 | 0.01 | 6 |
| 31241 | Diabetes monitoring admin.NOS | 7 | 0.00 | 6 |
| 34528 | Diabetes well being questionnaire | 1 | 0.00 | 6 |
| 36669 | Diabetic monitoring not required | 5 | 0.00 | 6 |
| 38130 | Diabetes wellbeing questionnaire | 3 | 0.00 | 6 |
| 47032 | Diabetes care plan agreed | 10 | 0.00 | 6 |
| 54846 | Diabetes monitoring deleted | 1 | 0.00 | 6 |
| 63412 | Diabetes clinical management plan | 3 | 0.00 | 6 |
| 7777 | Referral to diabetologist | 411 | 0.15 | 7 |
| 8306 | Referral to diabetes nurse | 115 | 0.04 | 7 |
| 8618 | Seen by diabetic liaison nurse | 25 | 0.01 | 7 |
| 9145 | DNA - Did not attend diabetic clinic | 94 | 0.04 | 7 |
| 11018 | Diabetic retinopathy 12 month review | 13 | 0.00 | 7 |
| 11041 | Excepted from diabetes qual indicators: Patient unsuitable | 262 | 0.10 | 7 |
| 11094 | Under care of diabetic foot screener | 229 | 0.09 | 7 |
| 11348 | Excepted from diabetes quality indicators: Informed dissent | 49 | 0.02 | 7 |
| 11930 | Under care of diabetes specialist nurse | 40 | 0.01 | 7 |
| 11977 | Referral to diabetes nurse | 59 | 0.02 | 7 |
| 12225 | Refer, diabetic liaison nurse | 224 | 0.08 | 7 |
| 12506 | Diabetes: practice programme | 470 | 0.18 | 7 |
| 12507 | Seen by diabetic liaison nurse | 16 | 0.01 | 7 |
| 12675 | Diabetes: shared care programme | 74 | 0.03 | 7 |
| 12682 | Patient offered diabetes structured education programme | 17 | 0.01 | 7 |
| 12703 | Education score - diabetes | 10 | 0.00 | 7 |
| 13057 | Health education - diabetes | 267 | 0.10 | 7 |
| 13678 | Referral to diabetic liaison nurse | 31 | 0.01 | 7 |
| 17886 | Diabetic - follow-up default | 10 | 0.00 | 7 |
| 18066 | Diabetic leaflet given | 54 | 0.02 | 7 |
| 18185 | Foot abnormality - non-diabetes | 6 | 0.00 | 7 |
| Medical code | Description | No. of patients | % | Categories |
| 18662 | Diabetic retinopathy 6 month review | 2 | 0.00 | 7 |
| 18766 | Diabetes resolved | 19 | 0.01 | 7 |
| 19381 | Referral to diabetic eye clinic | 10 | 0.00 | 7 |
| 26603 | Refuses diabetes monitoring | 3 | 0.00 | 7 |
| 26605 | Attended diabetes structured education programme | 2 | 0.00 | 7 |
| 26665 | Foot abnormality - non-diabetes | 4 | 0.00 | 7 |
| 28574 | Exception reporting: diabetes quality indicators | 11 | 0.00 | 7 |
| 28622 | Diabetes resolved | 18 | 0.01 | 7 |
| 28856 | Transition of diabetes care options discussed | 1 | 0.00 | 7 |
| 30648 | Did not attend diabetic retinopathy clinic | 12 | 0.00 | 7 |
| 32619 | Patient diabetes education review | 7 | 0.00 | 7 |
| 34450 | Hyperosmolar non-ketotic state in type 2 diabetes mellitus | 24 | 0.01 | 7 |
| 34541 | Private referral to diabetologist | 4 | 0.00 | 7 |
| 36633 | Hyperosmolar non-ketotic state in type 2 diabetes mellitus | 1 | 0.00 | 7 |
| 36855 | Retinal abnormality - non-diabetes | 1 | 0.00 | 7 |
| 45250 | Under care of diabetic liaison nurse | 2 | 0.00 | 7 |
| 46533 | Diabetic association member | 5 | 0.00 | 7 |
| 47011 | Referral to diabetes structured education programme | 51 | 0.02 | 7 |
| 47058 | Discharged from care of diabetes specialist nurse | 2 | 0.00 | 7 |
| 47341 | Diabetic crisis monitoring | 4 | 0.00 | 7 |
| 52237 | Patient held diabetic record issued | 12 | 0.00 | 7 |
| 54601 | Under care of diabetologist | 2 | 0.00 | 7 |
| 57389 | Patient consent given for addition to diabetic register | 7 | 0.00 | 7 |
| 57723 | Referral to diabetic register | 14 | 0.01 | 7 |
| 58639 | Patient held diabetic record declined | 1 | 0.00 | 7 |
| 61461 | Informed consent for diabetes national audit | 1 | 0.00 | 7 |
| 82474 | Referral to community diabetes specialist nurse | 45 | 0.02 | 7 |
| 83532 | Diabetes type 2 review | 58 | 0.02 | 7 |
| 93529 | DESMOND diabetes structured education programme completed | 1 | 0.00 | 7 |
| 93657 | Referral to DESMOND diabetes structured education programme | 45 | 0.02 | 7 |
| 93854 | Diabetes structured education programme declined | 1 | 0.00 | 7 |
| 93870 | Referral to XPERT diabetes structured education programme | 8 | 0.00 | 7 |
| 94330 | Referral to diabetes special interest general practitioner | 2 | 0.00 | 7 |
| 95159 | Did not attend DESMOND diabetes structured education program | 1 | 0.00 | 7 |
| 100436 | Education in self management of diabetes | 2 | 0.00 | 7 |
| 8414 | Pt advised re diabetic diet | 373 | 0.14 | 8 |
| 10642 | Dietary advice for diabetes mellitus | 192 | 0.07 | 8 |
| 13074 | Diabetic diet | 551 | 0.21 | 8 |
| 16881 | [V]Dietary counselling in diabetes mellitus | 25 | 0.01 | 8 |
| 21689 | Diabetic lipid lowering diet | 87 | 0.03 | 8 |
| Medical code | Description | No. of patients | % | Categories |
| 25041 | Dietary advice for type II diabetes | 2 | 0.00 | 8 |
| 25636 | Diabetic diet - poor compliance | 4 | 0.00 | 8 |
| 26604 | Diabetic diet - good compliance | 12 | 0.00 | 8 |
| 38078 | Understands diet - diabetes | 20 | 0.01 | 8 |
| 101801 | Type II diabetic dietary review | 1 | 0.00 | 8 |
| **Total** |  | **267,783** |  |  |

Categories:

1. T2DM specific
2. T2DM with complications
3. DM specific
4. DM with complications
5. Complication but no confirmed diagnosis mentioned, onset date likely to be earlier than this
6. Not confirmed diagnosis, but likely to be disease, so after true onset date
7. Other
8. Diet

**List A: Different classes of anti-diabetic medications**

1. Alphaglucosidase inhibitors
2. Biguanide/DP-4 inhibitor
3. Biguanide/Thiazolidinedione
4. Biguanides
5. DP-4 inhibitor
6. GLP-1 agonist
7. Meglitinide
8. Sulphonylureas
9. Thiazolidinedione
10. Insulin analogue
11. Animal insulin
12. Human insulin
13. Insulin (unknown)
